# Toll-Like Receptor Genes and Risk of Latent Tuberculosis Infection in People Infected with HIV-1

**DOI:** 10.3390/v16091371

**Published:** 2024-08-28

**Authors:** Svetlana Salamaikina, Ekaterina Kulabukhova, Vitaly Korchagin, Olga Khokhlova, Konstantin Mironov, Vasiliy Akimkin

**Affiliations:** 1 Central Research Institute of Epidemiology Federal Service for Surveillance on Consumer Rights Protection and Human Wellbeing Russian Federation, 111123 Moscow, Russia; 2 Medical Institute, The Peoples’ Friendship University of Russia (RUDN University), 117198 Moscow, Russia

**Keywords:** TLR, SNP, CD4 cells, expression, genetic susceptibility, HIV, latent tuberculosis infection

## Abstract

The purpose of this study was to determine the contribution of genetic factors, i.e., the level of expression and polymorphisms of Toll-like receptors (TLR), to the susceptibility of latent tuberculosis infection in a Russian cohort of individuals infected with HIV. The patients (*n* = 317) with confirmed HIV infection were divided into two groups according to the results of the STANDARD E TB-Feron test: 63 cases with a latent TB infection and 274 controls without LTBI. Total DNA and RNA were isolated from whole-blood samples. SNP genotyping and expression levels of five TLR genes (TLR1, TLR2, TLR4, TLR6, and TLR8) were determined by means of real-time PCR. There were no significant differences in the expression levels of the TLRs between the case and control groups. In addition, we did not observe any significant association between the analyzed SNPs and the susceptibility of Latent tuberculosis infection (LTBI) in patients with HIV. However, patients from an entire cohort with the rs4986790-GG (TLR4) and rs5743708-GG (TLR2) genotypes were characterized by lower CD4 T-cell counts compared to carriers of alternative alleles. Moreover, we found a significant risk of a hazardous drop in the CD4 T-cell count below 350 cells/mm^3^ associated with the rs4986790-G (TLR4) allele. Latent tuberculosis infection in individuals infected with HIV does not significantly modify the level of TLR gene expression.

## 1. Introduction

HIV and tuberculosis together form a lethal combination by accelerating each other’s progression. In 2022, the global tuberculosis incidence of HIV-positive patients was 671,000 (8.4 per 100,000 people), and about 167,000 people died from HIV-associated tuberculosis [1].

Latent tuberculosis infection (LTBI, TB infection) may reactivate to active tuberculosis when individuals are immunocompromised due to co-infection with HIV, the use of immunosuppressive drugs, diabetes mellitus, or other reasons [2,3,4]. There are no separate data on LTBI. According to the WHO, approximately 25% of the world’s population is infected with *Mycobacterium tuberculosis*, which also applies to people living with HIV who are 16 (uncertainty interval 14–18) times more likely to fall ill with tuberculosis disease than people without HIV. Tuberculosis is one of the leading causes of death among people infected with HIV.

The current diagnostic methods approved by the WHO for detecting *M. tuberculosis* antigens in patients with TB infection do not allow us to determine the phase of the patient’s condition and to differentiate it from tuberculosis disease [5]. It has been shown that gamma-interferon release assays are more sensitive and specific than skin tests, such as the Mantoux test and intradermal test with recombinant tuberculosis allergens (diaskintest), which are used for the diagnosis of tuberculosis infection. There are only three IGRA tests recommended by the WHO for detecting tuberculosis: the T-SPOT.TB spot test (Oxford Immunotec, Oxford, UK), QuantiFERON-TB Gold Plus (QFT-Plus, Qiagen, Germantown, MA, USA), and Wantai TB-IGRA (Wantai, Beijing, China) [6]. There are also some tests that are not yet recommended by the WHO but show good specificity and robustness, such as STANDARD TB-Feron IGRA (SD Biosensor, Inc., Suwon, Republic of Korea) [6].

Currently, research on genetic aspects of the host organism’s response to mycobacterial infection prior to the development of tuberculosis disease is becoming increasingly popular among researchers. However, there are practically no data available on the influence of genetic factors on LTBI among people infected with HIV.

Toll-like receptors (TLRs) are the primary receptors of the pathogen-associated molecular pattern (PAMP) system and play a crucial role in recognizing pathogens that enter the body. These receptors form the basis of the innate immune system and are able to detect ligands from bacteria, viruses, fungi, and protozoa. Activated TLRs trigger and regulate the intensity of the immune response, stimulating the production of pro-inflammatory cytokines, chemokines, and antimicrobial peptides. They also activate the adaptive immune system, helping to fight off infections. Each TLR is specialized in recognizing a specific type of pathogen, ensuring that the immune system can effectively respond to a wide range of threats. TLR2 and TLR4 are the main targets of host recognition of *M. tuberculosis* [7].

*TLR2*, expressed by antigen-presenting cells, plays an important role in the production of cytokines and chemokines that induce antimicrobial protection. However, HIV infection suppresses the expression of molecules involved in the *TLR2* signaling pathway (MyD88 and IRAK4), regardless of the presence of TLR2 in individuals with LTBI, which leads to a decrease in the production of cytokines necessary for controlling tuberculosis and preventing its progression [8].

The upregulation of specific TLR markers, especially TLR2 and TLR6, during latency may suggest the role these markers play in maintaining the continuous priming of the immune system and monocytes migrating to the LTBI site in the lungs, enabling them to better maintain local immunity [9].

TLR2 commonly forms a heterodimer with TLR1 and TLR6 and recognizes *M. tuberculosis* antigens such as *Rv0577* (TB27.3), *Rv2660c*, *Rv3875* (ESAT-6), *Rv3628*, *Rv2873* (MPT83), and *Rv1808* (PPE32). It leads to the activation of macrophages, NK-cells, CD4^+^T-cells, and DCs to produce cytokines to kill or maintain *M. tuberculosis* in the LTBI phase via a cascade reaction through the MyD88 pathway that upregulates the expression of genes [6]. An increase in TLR2 expression on CD14^low^ and CD14^high^ cells was observed in the peripheral blood of HIV-coinfected LTBI individuals compared to healthy HIV-negative, LTBI-positive individuals and active tuberculosis patients with or without HIV infection [8]. HIV infection prevents the production of TLR2-dependent pro-inflammatory cytokines and chemokines in individuals with LTBI [8]. Individuals carrying rs5743708 (*TLR2*) had a higher risk of tuberculosis development compared to the control group [6].

TLR4 recognizes *M. tuberculosis* antigens such as *Rv3478* (PPE60), *Rv3417* (groEL1), *Rv0440* (groEL2), *Rv0652* (RplL), *Rv0475* (HBHA), *Rv1009* (RpfB), and 38 KD glycoprotein, which activate macrophages, DCs, and Th1 cells to secrete pro-inflammatory cytokines [6]. *TLR4* expression was significantly increased in patients with tuberculosis, HIV, and tuberculosis–HIV coinfection compared to the control group [10]. *TLR4* expression was equally increased on classical and intermediate monocytes, while *TLR2* expression was higher on intermediate than classical monocytes [10]. *TLR4* expression positively correlated with plasma biomarkers, while *TLR2* had an inverse correlation. *TLR4* is associated with the severity of the disease, and *TLR2* is associated with the immunocompetent status of patients [10]. *TLR4* stimulation has been shown to be associated with reduced odds of tuberculosis relapse in ART-treated patients [11].

The aim of this study was to determine the contribution of genetic factors, namely the level of expression and polymorphisms of TLR, to the development of LTBI in people infected with HIV.

In previous work, we focused on the association between single-nucleotide polymorphisms (SNPs) and the risk of tuberculosis in people living with HIV. We found significant differences in allele frequencies between the HIV and HIV–tuberculosis groups [12,13].

The polymorphisms we studied earlier are located in the regulatory regions of genes (Table 1); therefore, it is important to study the expression level in combination with allele frequencies in samples of patients infected with HIV [14].

## 2. Materials and Methods

### 2.1. Ethics Approval

This study was conducted according to the guidelines of the Declaration of Helsinki and approved by the Ethics Committee of the Central Research Institute of the Epidemiology Federal Service for Surveillance on Consumer Rights Protection and Human Wellbeing Russian Federation (protocol code No. 136 dated 25 May 2023).

### 2.2. Clinicopathological Data of Selected Patients

Depersonalized samples from patients with HIV-1 (*n* = 395) used in this study were collected in infectious disease clinics of the Central Research Institute of Epidemiology (CRIE) in 2023. All patients had subclinical HIV infection and had been on antiretroviral therapy for at least two years at the time of inclusion in this study. All patients received standard antiretroviral therapy regimens according to the protocol adopted in Russia based on the WHO guidelines [15]. Most of the study subjects received treatments based on non-nucleoside reverse transcriptase inhibitors developed and approved for the treatment of HIV infection in Russia [16].

HIV was diagnosed in accordance with country-specific guidelines based on the WHO guidelines. This diagnosis was made on the basis of two positive ELISA tests confirmed by immunoblot [17,18].

LTBI was diagnosed with STANDARD TB-Feron IGRA (SD Biosensor, Inc., Gyeonggi-Do, Republic of Korea). The assay was performed according to the manufacturer’s instructions. Briefly, one milliliter of whole blood was added to each of the three TB-Feron tubes (a negative control tube, a TB antigen tube, and a tube with the mitogen-positive control). The tubes were treated as recommended by the manufacturer, the IFN-γ concentration (IU/mL) in plasma was measured by an ELISA reader, and the LTBI status was calculated by ‘TB-Feron IGRA—analysis Software’(version 1.4.3) (SD Biosensor, Inc., Gyeonggi-Do, Republic of Korea).

The inclusion criteria for patients with HIV were confirmed diagnosis of HIV infection, data regarding CD4 T-cell count, viral load at the time of inclusion in this study (no more than 6 months of follow-up), and no history of active tuberculosis.

The inclusion criteria for patients coinfected with HIV and LTBI were confirmed diagnosis of HIV infection, positive result of the STANDARD TB-Feron IGRA (SD Biosensor, Inc., Gyeonggi-Do, Republic of Korea) test, data regarding CD4 T-cell count, and viral load at the time of inclusion in this study (no more than 6 months of follow-up).

The exclusion criteria were acute HIV infection, tuberculosis or any other infectious disease progression, and pathological changes detected by X-ray analysis that was performed after receiving a positive STANDARD TB-Feron IGRA (SD Biosensor, Inc., Gyeonggi-Do, Republic of Korea) test.

In summary, there were 276 patients infected with HIV-1 included in the control group and 63 patients infected with HIV-LTBI in the case group. There were no differences between groups in terms of gender and age (Table 2).

### 2.3. CD4 T-Cell Counts and Relative Number of Classical, Inflammatory, and Non-Classical Monocyte Measurements

CD4 T-cell counts were obtained via the flow cytofluorimeter FACSCalibur (Becton Dickinson, Franklin Lakes, NJ, USA) according to the standard manufacturer’s protocols.

Human CD45/CD14, IgG1/IgG2a, FITC/PE (Beckman Coulter, Brea, CA, USA), and CD16 and Mouse IgG1, 3G8, and PC5 (Beckman Coulter, Brea, CA, USA) were used for flow cytometry. Measurements of the relative number of classical, inflammatory, and non-classical monocytes (CD14^+^CD16^−^, CD14^+^CD16^+^, and CD14^low^CD16^+^) were performed on a Navios TM (Beckman Coulter, Brea, CA, USA) flow cytometer.

Reference values for the measured cell groups were taken as 78.83–90.37 for the classical monocyte group (CD14^+^CD16^−^), 2.5–9.3 for the inflammatory monocyte group (CD14^+^CD16^+^), and 5.77–13.25 for the non-classical monocyte group (CD14^low^CD16^+^).

### 2.4. DNA and mRNA Extraction

Peripheral venous blood samples anticoagulated by EDTA from every participant were stored at 4 °C. Genomic DNA was isolated from collected blood samples using a RIBO-prep kit (AmpliSens, Moscow, Russia) following the manufacturer’s protocol.

The isolated mRNA samples were evaluated in the RNA-carrying buffer and were stored at −20 °C for no more than a week before use.

### 2.5. SNP Selection and Allele Detection

The SNPs used in this study were selected and described in our previous work [13]. Real-time PCR primers and probes were designed for appropriate alleles using reference sequences from the SNP database [19]. We followed the design guidelines according to real-time recommendations from the instrument manufacturer (Qiagen, Hamburg, Germany) [20]. The annealing temperature for the probes was adjusted to 60 °C. The concentrations of primers and probes were chosen empirically and ranged from 0.2 to 0.4 and from 0.06 to 0.12 µM, respectively. The designed oligonucleotide sequences have been presented previously [13].

PCR reactions were carried out in a final volume of 25 μL, containing 10 μL of extracted DNA, 10 μL of dNTPs (0.44 mM), primers, probes, 4.5 μL of RT-PCR-mix-2 FEP/FRT reagents, and 0.5 μL of TaqF-polymerase. The real-time PCR amplification was performed using the Rotor Gene Q cycler (Qiagen, Hamburg, Germany) with the following procedure: 95 °C for 15 min (1 cycle) and then 95 °C for 5 s, 60 °C for 20 s, and 72 °C for 10 s (45 cycles with fluorescent signal detection at 60 °C). In the amplification results analysis, the threshold was set at 10% of the highest fluorescent signal value for each channel.

The confirmation of the real-time PCR results was performed by PyroMark Q24 sequencing using the reagent kits recommended by the equipment manufacturer (Qiagen, Hamburg, Germany).

### 2.6. Expression Level Detection by One-Step Real-Time Multiplex PCR

Housekeeping genes that, according to literature data, maintain a stable level of expression in peripheral blood were used as reference genes to control the expression level: hypoxanthine guanine phosphoribosyltransferase (*HPRT1*), succinate dehydrogenase complex, subunit A (*SDHA*), glyceraldehyde-3-phosphate dehydrogenase (*GAPDH*), and transcription factor IID binding protein TATA-box protein (*TBP*) [21,22,23,24]. The *TLR1*, *TLR2*, *TLR4*, *TLR6,* and *TLR8* genes (target genes) were selected for expression determination. The designed oligonucleotide sequences are presented in Supplementary Table S1.

The concentrations of primers and probes were chosen empirically and ranged from 0.2 to 0.4 and 0.06 to 0.12 μM, respectively. The idtDNA calculator [25] was used to select the melting temperature; the specificity of primers for DNA and RNA matrices was checked using the Primer-BLAST resource [26].

A pair of primers and a fluorescently labeled probe were selected for each gene fragment. The primer sequences were chosen to be specific only to active splice variants characterized in the NCBI database. The design of oligonucleotides was made in such a way as to exclude DNA amplification due to either the annealing of one of the primers simultaneously in two neighboring exons or the mutual arrangement of primers in exons separated by an intron of several thousand base pairs, which does not allow the product of DNA amplification to be obtained using TaqF-polymerase.

The real-time PCR amplification was performed using a Bio-Rad CFX96 cycler (Bio-Rad, Hercules, CA, USA) with the following procedure: 50 °C for 15 min (1 cycle); 95 °C for 15 min (1 cycle); and 95 °C for 5 s, 60 °C for 20 s, and 72 °C for 10 s (45 cycles with fluorescent signal detection at 60 °C). Ct determination modes were set as a single threshold and calculated automatically by program settings for the amplification system. The mean Ct values for each gene were calculated as the geometric mean (GM [Ct]) of independent repeats.

The geNorm [27] and BestKeeper [28] algorithms implemented in the ctrlGene package [29] were used to determine the stability of the expression of housekeeping genes. The average expression stability (M) values of the analyzed genes were calculated in geNorm by stepwise exclusion of the least stably expressed genes in each round until the genes with the most stable expression (lowest M values) remained. BestKeeper calculates descriptive statistics based on the Ct data and pairwise correlation between all analyzed genes, taking into account the standard deviation (SD) of the Ct values, where the lowest value represents the gene with the most stable expression in the ranking index. Based on the Ct values of the most stably expressed genes, BestKeeper indices were calculated for each sample and used further for normalization in the analysis of target gene expression.

The ratio of (GM[Ct]) to the BestKeeper index calculated on the basis of the most stable housekeeping genes was used as a normalized index of target gene expression (dCt). To characterize the dispersion of dCt values, descriptive statistics and scatter plots were calculated for each gene.

### 2.7. Statistical Analysis

Real-time RT-PCR results were preliminarily processed in Microsoft Excel. Samples with a positive signal in at least one repeat out of three were selected. The matrix of Ct values and SNP genotypes was exported to the R environment (version 4.4.1) [30]. We used Pearson’s χ^2^ test for the analysis of contingency tables and the nonparametric Mann–Whitney U test for between-group comparisons. Association and risk scores (ORs) were determined using SNPassoc [31] packages according to prespecified genetic risk models—codominant, dominant, recessive, overdominant, and log-additive [32,33]. The results of the statistical analysis were visualized in the form of diagrams using the ggplot2 and ggstatsplot packages [34,35]. A value of *p* < 0.05 was used as the criterion for statistical significance of the results. Holm–Bonferroni correction was used for multiple comparisons.

## 3. Results

### 3.1. Toll-Like Receptor Gene Expression

We used housekeeping genes (*HPRT1*, *SDHA*, *GAPDH,* and *TBP*) to normalize the expression level of the TLR genes by one-step multiplex real-time PCR. The expression of all the housekeeping genes involved in the study sample demonstrates high stability. The GM[Ct] value comparison revealed the highest expression level of the *GAPDH* gene with a mean value of 21.5 (2.5), whereas the highest mean GM[Ct] value (lowest expression) was observed for *HPRT1* (28.5(1.9)). The calculation of expression stability using geNorm showed that the *HPRT1* and *SDHA* pair had the highest stability among all genes—their lowest M value was 1.60 (1.82 and 1.97 for *GAPDH* and *TBP*, respectively). The analysis of the Ct values of the TLR genes showed that *TLR6* is characterized by the lowest expression (GM[Ct] = 29.3(2.4)) and lags behind the others by an average of 3.8 cycles.

**Figure 1:**
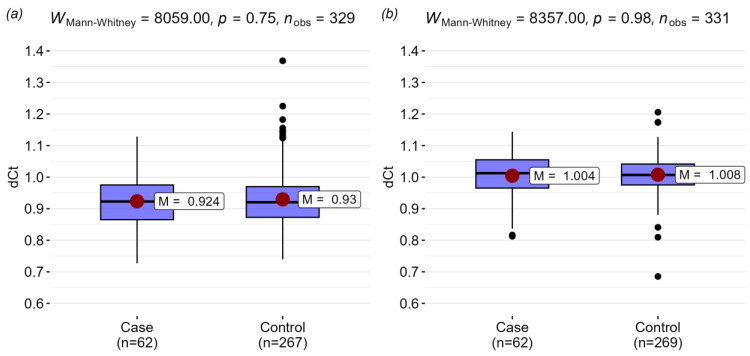

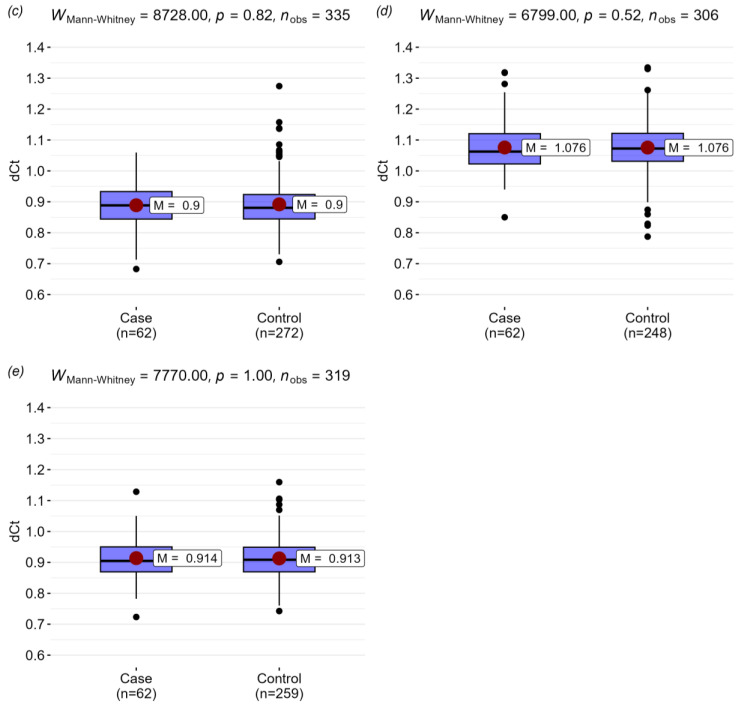
TLR gene expression comparison between case and control groups: (**a**) *TLR1*; (**b**) *TLR2*; (**c**) *TLR4*; (**d**) *TLR6*; (**e**) TLR8 (M denotes mean dCT value).

The expression level in the study group correlated with neither the age, CD4 T-cell count, leukocytes, and lymphocytes nor the relative number of classical, inflammatory, and non-classical monocytes (CD14^+^CD16^−^, CD14^+^CD16^+^, and CD14^low^CD16^+^). The expression level of the *TLR1*, *TLR2*, *TLR4*, *TLR6,* and *TLR8* genes in the case (*n* = 63) and control (*n* = 274) groups did not show statistically significant differences (Figure 1).

### 3.2. TLR Gene Polymorphism Association with Latent Tuberculosis Coinfection

Six candidate SNPs in five TLR genes were analyzed: rs5743551 (*TLR1*), rs5743708 (*TLR2*), rs3804100 (*TLR2*), rs4986790 (*TLR4*), rs5743810 (*TLR6*), and rs3764880 (*TLR8*).

We did not observe any significant association between LTBI in patients with HIV and any of the analyzed SNPs. Estimates for the models for each SNP are presented in Supplementary Table S2.

**Figure 2:**
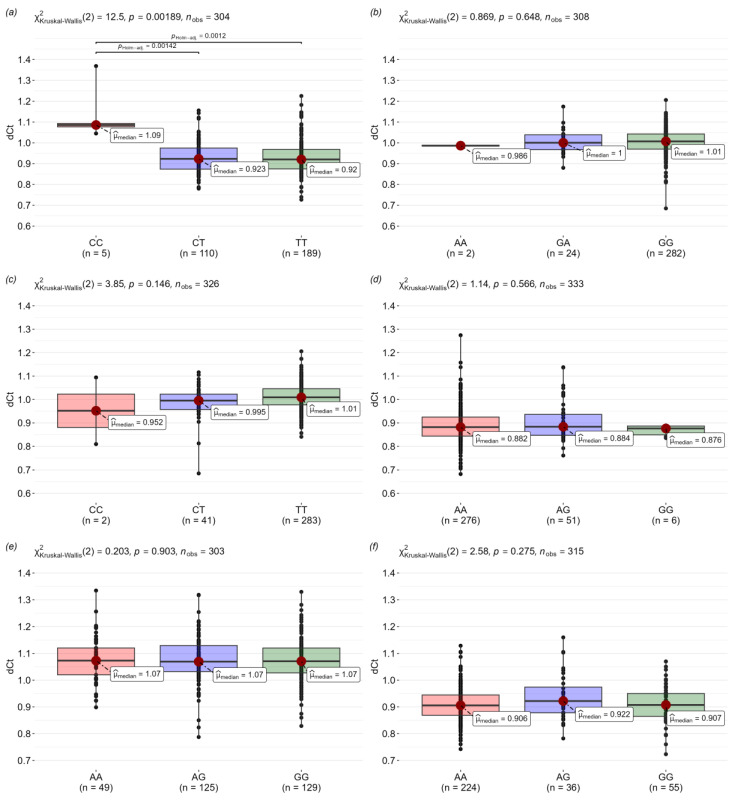
The distribution of gene expression in relation to genotype: (**a**) rs5743551 (*TLR1*); (**b**) rs5743708 (*TLR2*); (**c**) rs3804100 (*TLR2*); (**d**) rs4986790 (*TLR4*); (**e**) rs5743810 (*TLR6*); and (**f**) rs3764880 (*TLR8*).

We also analyzed the correlation between SNP genotypes and expression level (Figure 2). There is a trend towards a lower expression level in patients with the rs5743551-CC genotype in comparison to the rs5743551-CT and rs5743551-TT genotypes in the *TLR1* gene. Genotypes studied at the SNP loci in the *TLR2*, *TLR4*, *TLR6*, and *TLR8* genes were not significantly different in expression level.

### 3.3. Association between TLR Gene Polymorphisms and Changes in CD4, CD14, and CD16 T-Cell Count in Individuals Positive for HIV

**Figure 3:**
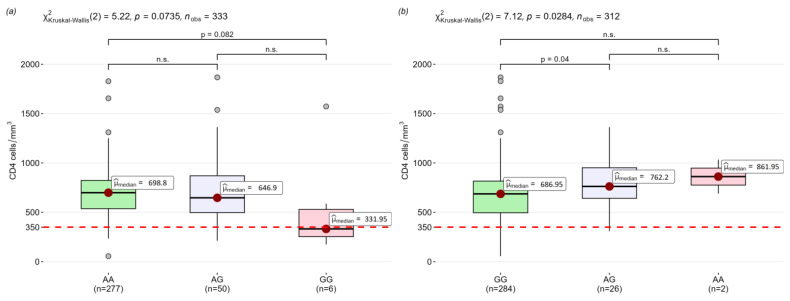
Differences in CD4 T-cell count between genotypes of (**a**) rs4986790 (*TLR4*) and (**b**) rs5743708 (*TLR2*); n.s.= non-significant.

In previous studies, we observed the correlation between CD4 T-cell count and genotypes for patients coinfected with tuberculosis and HIV [13]. In the present study, we found no significant differences in T-cell counts between the case and control groups in CD4, classical (CD14^+^CD16^−^), inflammatory (CD14^+^CD16^+^), and non-classical monocytes (CD14^low^CD16^+^), so we performed genotype association analyses for polymorphic loci on a sample that included both groups. A tendency was identified for the association of loci rs4986790 (*TLR4*) and rs5743708 (*TLR2*) with a lower CD4 T-cell count in patients carrying the rs4986790-GG and rs5743708-GG genotypes (Figure 3).

We compared the groups with high (more than 350 cells per mm^3^) and low (less than 350 cells per mm^3^) CD4 T-cell counts (Supplementary Table S3). The selected results of the analyses are presented in Table 3. Although in the previous step we observed no statistically significant differences between the rs4986790 (*TLR4*) genotypes in the CD4 T-cell count, we found a significant risk for the GG and AG genotypes to reduce the CD4 T-cell count below 350 cells/mm^3^. In contrast, the CD4 T-cell counts in groups with GG and AG genotypes of the rs5743708 (*TLR2*) loci were in the range of concentrations over 350 cells/mm^3^, so we did not find an association of these genotypes with the risk of a drop in CD4 T-cell count below 350 cells/mm^3^.

## 4. Discussion

The global prevalence of LTBI was found to be 24.8% [36]. In particular, Southeast Asia emerged as the region with the highest LTBI prevalence of 27.7%, followed by Africa at 26.6% [37]. In our study, it was 18.8% (63/335). The data observed in our study are congruent with the prevalence in other countries: Anne Bourgarit et al. reported an LTBI prevalence of 11.5% (47/407, France), Helena A. White et al. reported a prevalence of 11.1% (117/1053, United Kingdom), and Tessa Runels et al. reported a prevalence of 5.8% (11/189, United States of America) [38,39,40].

Host immune response receptors such as TLRs are used to recognize mycobacterial infection. In the present study, we investigated the association of the expression and single-nucleotide polymorphisms of TLR genes with LTBI in patients infected with HIV. We found no differences between the case (positive IGRA test to M. tuberculosis) and control (infected with HIV without *M. tuberculosis* coinfection) groups in any of the assessed parameters, which may indicate that the antiretroviral therapy (ART) taken by all the patients allows the development of opportunistic infections to be suppressed. In the absence of differences, we performed all subsequent comparisons on samples that included all individuals studied who were infected with HIV.

In a previous study, we found an association of the rs4986790 (*TLR4*) and rs5743810 (*TLR6*) polymorphisms with the risk of developing tuberculosis [13]. The lack of such an association in the present study may be due to the following reasons: the samples were recruited in different periods, in different countries, and we only observed an active tuberculosis infection status in the previous study.

Here, we additionally analyzed the correlation between the studied genotypes and the expression level of the TLR genes. No significant differences were found, but there was a tendency towards a lower expression level in patients with the rs5743551-CC genotype in comparison with the rs5743551-CT and rs5743551-TT genotypes in the *TLR1* gene. However, this trend does not reach statistical significance due to the rarity of the rs5743551-CC genotype in the study sample.

In our previous study, we also found an association between the rs4986790-G (*TLR4*) allele and increased CD4 T-cell count in the group coinfected with HIV and tuberculosis [13]. We hypothesized that the correlations we observed might be related to TLR gene expression, but this assumption was not confirmed in the present study. We observed a trend towards lower CD4 T-cell counts in patients with the rs4986790-GG (*TLR4*) and rs5743708-GG (*TLR2*) genotypes, which is consistent with the results obtained in a previous study in a group of individuals positive for HIV not coinfected with *M. tuberculosis*. The lack of association of genotypes with the differences in expression levels may indicate the linkage of these loci with an attenuated immune response to LPS and lower secretion levels of pro-inflammatory cytokines [41,42,43].

In addition to CD4 T-cells, HIV infects monocytes that express CD14^+^CD16^+^, which contributes to the formation, dispersal, and maintenance of viral reservoirs [44,45,46,47,48]. A comparison of the transcriptome of PBMCs from patients with HIV-1 infection and those with HIV-1 and tuberculosis coinfection revealed a higher proportion of the inflammatory CD14^+^CD16^+^ monocyte subset in the HIV and tuberculosis coinfection cohort [11]. Leon-Rivera et al. used scRNA-seq to simultaneously detect HIV, host HIV-infected transcripts, and HIV-exposed monocytes in both ART-treated and non-ART-treated patients [49]. Monocyte subsets provide varying degrees of cellular milieu permissive to HIV infection, and ART has a detrimental effect on monocyte function [49]. Therefore, it was important for us to study the association between the polymorphisms and the level of expression of the TLR genes with monocytic cell counts. Unfortunately, we were unable to detect significant associations, which may be due to the absence of an active infectious process or the presence of long ART treatment in the studied patients.

Polymorphic loci rs5743708 (*TLR2*) influence the risk of developing LTBI and subsequent pulmonary tuberculosis in the Chinese population, and variations in rs7873784 (*TLR4*) and rs5743836 (*TLR9*) influence the risk of developing LTBI and pulmonary tuberculosis [50]. In the present study, we do not observe such correlations with the studied genotypes in the *TLR2* and *TLR4* genes, which may be due to population characteristics. Epigenetic regulation of gene expression could be one of the potential risk factors for the progression of LTBI to active tuberculosis. The methylation of certain CpG sites in the *TLR2* promoter has been shown to reduce expression levels in natural killer cells/monocytes of patients with active pulmonary tuberculosis and correlates with bacterial load and disease severity [51]. Thus, DNA hypermethylation in the monocytes may indicate severe disease [52] and can be used for the opportune chemoprevention of tuberculosis.

Epidemiological, basic, and genetic studies will expand our understanding of the key factors that determine host susceptibility to *M. tuberculosis* in different populations and may provide new insights into the influence of genetic heterogeneity on the development of tuberculosis infection.

## 5. Conclusions

According to our findings, there was no correlation between expression level and carrying LTBI in patients infected with HIV that were included in our study. We did not observe any significant association between LTBI in patients with HIV and any of the analyzed SNPs after multiple test correction. There was a tendency of association of the loci rs4986790 (*TLR4*) and rs5743708 (*TLR2*) with a lower CD4 T-cell count in patients carrying the rs4986790-GG and rs5743708-GG genotypes. It is possible that the suggested SNPs we studied contribute to the expression level of other genes. LTBI in individuals infected with HIV does not significantly modify the level of TLR gene expression. Therefore, additional work is necessary, which could be conducted in the future.

## Figures and Tables

**Table 1:** Single-nucleotide polymorphisms in TLR genes used in previous studies.

Gene	Position (GRCh38p.14)	RefSNP ID	Gene Location
*TLR1*	Chr 4:38806033	rs5743551	intron variant
			promoter region
*TLR2*	Chr 4:153705165	rs5743708	exon variant
		rs3804100	exon variant
*TLR4*	Chr 9:117713024	rs4986790	exon variant
*TLR6*	Chr 4:38828729	rs5743810	exon variant
*TLR8*	Chr X:12906707	rs3764880	intron variant
			start codon

**Table 2:** Demographic characteristics of study samples.

	Case	Control	Statistical Criterion
Gender, *n* (%):			Pearson’s Chi-Squared test, *p* = 0.361
Male	46 (73)	181 (66)	
Female	17 (27)	93 (34)	
Age, year:			Mann–Whitney test, *p* = 0.373
Mean (sd)	43.4 (8.85)	42.2 (8.21)	

**Table 3:** Association between TLR gene polymorphisms and crucial changes in CD4 T-cell count in patients with HIV.

SNP	“CD4 T-Cell High”, *n* (%)	“CD4 T-Cell Low”, *n* (%)	Median Conc. (cells/mm^3^)	OR (CI 95%)	*p*
rs4986790 (*TLR4*)					
A/A	267 (86)	11 (48)	698.8	1.00	6.99×10^−5^
A/G	42 (13)	9 (39)	646.9	5.20 (2.03–13.30)	
G/G	3 (1)	3 (13)	331.95	24.27 (4.39–134.22)	
rs5743708 (*TLR2*)					
A/A	264 (90.7)	22 (96)	861.95	1.00	0.7476
A/G	25 (8.6)	1 (4)	762.2	0.48 (0.06–3.71)	
G/G	2 (0.7)	0	686.95	0.00	

## Data Availability

The original data presented in the study are openly available in FigShare at [https://doi.org/10.6084/m9.figshare.26411299.v1] https://figshare.com/s/8002a7a8e831248f78d7 accessed on 30 July 2024.

## Supplementary Materials

The following supporting information can be downloaded at https://www.mdpi.com/article/10.3390/v16091371/s1. Table S1: Analyzed SNP association with LTBI in patients with HIV; Table S2: Association between TLR gene polymorphism and crucial changes in CD4 T-cell count in patients with HIV; Table S3: Oligonucleotide sequences for measuring expression level in TLR genes.
