# Supplementary material for: Toll-Like Receptor Genes and Risk of Latent Tuberculosis Infection in People Infected with HIV-1
Source: Viruses. 2024 Aug 28;16(9):1371. doi: 10.3390/v16091371 (PMC11436194; doi:10.3390/v16091371)
Supplement: Supplementary file 1 [file viruses-16-01371-s001.zip › viruses-3158002-supplementary.pdf]

Table S1. Analyzed SNPs association with LTBI in HIV patients.

SNP	Control	Allele 1 freq	Case	Allele 2 freq	OR CI95%	*p*
rs3764880 (*TLR8*)						
Codominant						
A/A	193	70.4	46	75.4	1.00	0.7284
A/G	31	11.3	6	9.8	0.81 (0.32-2.06)	
G/G	50	18.2	9	14.8	0.76 (0.35-1.65)	
Dominant						
A/A	193	70.4	46	75.4	1.00	0.4318
A/G-G/G	81	29.6	15	24.6	0.78 (0.41-1.47)	
Recessive						
A/A-A/G	224	81.8	52	85.2	1.00	0.5095
G/G	50	18.2	9	14.8	0.78 (0.36-1.68)	
Overdominant						
A/A-G/G	243	88.7	55	90.2	1.00	0.7358
A/G	31	11.3	6	9.8	0.86 (0.34-2.15)	
log-Additive						
0,1,2	274	81.8	61	18.2	0.86 (0.59-1.26)	0.4332
rs4986790 (*TLR4*)						
Codominant						
A/A	224	81.5	55	88.7	1.00	0.3404
A/G	46	16.7	6	9.7	0.53 (0.22-1.31)	
G/G	5	1.8	1	1.6	0.81 (0.09-7.11)	
Dominant						
A/A	224	81.5	55	88.7	1.00	0.154
A/G-G/G	51	18.5	7	11.3	0.56 (0.24-1.30)	
Recessive						
A/A-A/G	270	98.2	61	98.4	1.00	0.911
G/G	5	1.8	1	1.6	0.89 (0.10-7.71)	
Overdominant						
A/A-G/G	229	83.3	56	90.3	1.00	0.1455
A/G	46	16.7	6	9.7	0.53 (0.22-1.31)	
log-Additive						
0,1,2	275	81.6	62	18.4	0.63 (0.30-1.33)	0.2013
rs5743551 (*TLR1*)						
Codominant						
T/T	158	62.2	39	65.0	1.00	0.4571
C/T	93	36.6	19	31.7	0.83 (0.45-1.52)	
C/C	3	1.2	2	3.3	2.70 (0.44-16.72)	
Dominant						
T/T	158	62.2	39	65.0	1.00	0.6861
C/T-C/C	96	37.8	21	35.0	0.89 (0.49-1.60)	
Recessive						
T/T-C/T	251	98.8	58	96.7	1.00	0.2763
C/C	3	1.2	2	3.3	2.89 (0.47-17.66)	
Overdominant						
T/T-C/C	161	63.4	41	68.3	1.00	0.4687
C/T	93	36.6	19	31.7	0.80 (0.44-1.46)	

log-Additive						
0,1,2	254	80.9	60	19.1	0.98 (0.57-1.68)	0.9312
rs5743810 (*TLR6*)						
Codominant						
G/G	109	39.8	30	48.4	1.00	
A/G	117	42.7	26	41.9	0.81 (0.45-1.45)	0.2161
A/A	48	17.5	6	9.7	0.45 (0.18-1.16)	
Dominant						
G/G	109	39.8	30	48.4	1.00	0.2163
A/G-A/A	165	60.2	32	51.6	0.70 (0.41-1.23)	
Recessive						
G/G-A/G	226	82.5	56	90.3	1.00	0.1102
A/A	48	17.5	6	9.7	0.50 (0.21-1.24)	
Overdominant						
G/G-A/A	157	57.3	36	58.1	1.00	0.9123
A/G	117	42.7	26	41.9	0.97 (0.55-1.69)	
log-Additive						
0,1,2	274	81.5	62	18.5	0.71(0.48-1.07)	0.0974
rs5743708 (*TLR2*)						
Codominant						
G/G	234	91.4	54	90	1.00	
G/A	20	7.8	6	10	1.30 (0.50-3.39)	0.7401
A/A	2	0.8	0	0	-	
Dominant						
G/G	234	91.4	54	90	1.00	0.7337
G/A-A/A	22	8.6	6	10	1.18 (0.46-3.06)	
Overdominant						
G/G-A/A	236	92.2	54	90	1.00	0.5875
G/A	20	7.8	6	10	1.31 (0.50-3.42)	
log-Additive						
0,1,2	256	81.0	60	19	1.06 (0.44-2.56)	0.7401
rs3804100 (*TLR2*)						
Codominant						
T/T	238	87.2	52	85.2	1.00	
C/T	33	12.1	9	14.8	1.25 (0.56-2.77)	0.7793
C/C	2	0.7	0	0.0	-	
Dominant						
T/T	238	87.2	52	85.2	1.00	0.6901
C/T-C/C	35	12.8	9	14.8	1.18 (0.53-2.60)	
Overdominant						
T/T-C/C	240	87.9	52	85.2	1.00	0.577
C/T	33	12.1	9	14.8	1.26 (0.57-2.79)	
log-Additive						
0,1,2	273	81.7	61	18.3	1.09 (0.52-2.31)	0.7793

Table S2. Association TLR gene polymorphism with crucial changes in CD4 T-cell count in patients with HIV.

SNP	Control (>350 cells/mm^3)	Allele1 freq	Case (<350 cells/mm^3)	Allele2 freq	OR (CI95%)	p ($p_{adjust*}$)

rs3764880 (*TLR8*)						
Codominant						
A/A	223	71.7	14	63.6	1.00	0.7301
A/G	34	10.9	3	13.6	1.41 (0.38-5.15)	
G/G	54	17.4	5	22.7	1.47 (0.51-4.27)	
Dominant						
A/A	223	71.7	14	63.6	1.00	0.4292
A/G-G/G	88	28.3	8	36.4	1.45 (0.59-3.57)	
Recessive						
A/A-A/G	257	82.6	17	77.3	1.00	0.5369
G/G	54	17.4	5	22.7	1.40 (0.50-3.96)	
log-Additive						
0,1,2	311	93.4	22	6.6	1.23 (0.73-2.06)	0.4457
rs4986790 (*TLR4*)						
Codominant						
A/A	267	85.6	11	47.8	1.00	0.00007 (0.00042)
A/G	42	13.5	9	39.1	5.20 (2.03-13.30)	
G/G	3	1.0	3	13.0	24.27 (4.39-134.22)	
Dominant						
A/A	267	85.6	11	47.8	1.00	0.000054 (0.00032)
A/G-G/G	45	14.4	12	52.2	6.47 (2.69-15.56)	
Recessive						
A/A-A/G	309	99.0	20	87.0	1.00	0.00353 (0.021)
G/G	3	1.0	3	13.0	15.45 (2.93-81.50)	
log-Additive						
0,1,2	312	93.1	23	6.9	5.05 (2.51-10.16)	0.000012 (0.000072)
rs5743551 (*TLR1*)						
Codominant						
T/T	181	62.6	15	65.2	1.00	1
C/T	103	35.6	8	34.8	0.94 (0.38-2.29)	
C/C	5	1.7	0	0.0	-	
Dominant						
T/T	181	62.6	15	65.2	1.00	0.804
C/T-C/C	108	37.4	8	34.8	0.89 (0.37-2.18)	
log-Additive						
0,1,2	289	92.6	23	7.4	0.85(0.36-1.97)	1
rs5743810 (*TLR6*)						
Codominant						
G/G	130	41.9	8	33.3	1.00	0.6664
A/G	131	42.3	11	45.8	1.36 (0.53-3.50)	
A/A	49	15.8	5	20.8	1.66 (0.52-5.31)	
Dominant						
G/G	130	41.9	8	33.3	1.00	0.4044
A/G-A/A	180	58.1	16	66.7	1.44 (0.60-3.48)	
Recessive						
G/G-A/G	261	84.2	19	79.2	1.00	0.5326
A/A	49	15.8	5	20.8	1.40 (0.50-3.93)	
Overdominant						
G/G-A/A	179	57.7	13	54.2	1.00	0.7336

A/G	131	42.3	11	45.8	1.16 (0.50-2.66)	
log-Additive						
0,1,2	310	92.8	24	7.2	1.30 (0.73-2.29)	0.3729
rs5743708 (*TLR2*)						
Codominant						
G/G	264	90.7	22	95.7	1.00	0.7476
G/A	25	8.6	1	4.3	0.48 (0.06-3.71)	
A/A	2	0.7	0	0.0	-	
Dominant						
G/G	264	90.7	22	95.7	1.00	0.3823
G/A-A/A	27	9.3	1	4.3	0.44 (0.06-3.42)	
log-Additive						
0,1,2	291	92.7	23	7.3	0.45 (0.06-3.22)	0.7476
rs3804100 (*TLR2*)						
Codominant						
T/T	265	86.0	23	95.8	1.00	0.4287
C/T	41	13.3	1	4.2	0.28 (0.04-2.14)	
C/C	2	0.6	0	0.0	-	
Dominant						
T/T	265	86.0	23	95.8	1.00	0.1213
C/T-C/C	43	14.0	1	4.2	0.27 (0.04-2.04)	
log-Additive						
0,1,2	308	92.8	24	7.2	0.27 (0.04-2.02)	0.4287

*p adjustment by Holm-Bonferroni.

Table S3. Oligonucleotide sequences for measuring expression level in TLR genes.

**Oligonucleotide**	**5′-3′ sequence**
E TLR1-F	TGGCAAAATGGAAGATGCTAGTC
E TLR1-R	AGACTGCCAAATGGAACAGACAA
TLR1-Z3	(ROX)CCC GGA GGC AAT(BHQ1)GCT GCT GTT CAG CTC
TLR2-F	AGG CAG CGA GAA AGC GCA GC
TLR2-R	CCTTGGAGAGGCTGATGATGA
TLR2-Z3	(Cy5.5)CCCCCAAGACCCACACCATCCACAAAG(BHQ1)
TLR4-F	TTTCCCAGAACTGCAGGTGCTG
TLR4-R	CTCTGGATGGGGTTTCCTGTCA
TLR4-Z2	(R6G)GGGGCATATCAGAGCCT(BHQ1)AAGCCACCTCTCT
TLR6-F	ATGATGTTGCAGTGGCTATCCTAA
TLR6-R1-2	CAACTCAGAGTTTTCACTCTTCCAAAG
TLR6-Z3	(Cy5)GGGTTGTTCTTCT(BHQ1)TCAGAGCATCTTGATATGAGTCC

TLR8-F	AGTTTCTCTTCTCGGCCACCT
TLR8-R	AGCAGGAAAATGCAGGTCAGC
TLR8-Z3	(Cy5)CCT GCA TAG AGG GTA CCA TTC TGC GCT GCT GC(BHQ1)
HPRT1-FII	TTgCTTTCCTTggTCAggCA
HPRT1-RII	CCAACAAAgTCTggCTTATATCCAAC
HPRT1-ZII-1	(FAM)CgTggggTCCTTTTCACCAgCAAgC(BHQ1)
SDHA-F	GCGGCAACAGCAGACATGT
SDHA-R2	CTGTTTGCAACACTGTTGGCC
SDHA-Z1	(R6G)CCGGGGCCTGTCGCGGCTGCTG(BHQ1)
GAPDH-F2	GAAGGACTCATGCACCACGGTC
GAPDH-R	GCAGGGATGATGTTCTGGAGA
GAPDH-Z	(ROX)GCCCCGCGGCCATCACGCCACAG(BHQ)
TBP-F	ggTTTgCTgCggTAATCATgA
TBP-R	CAgTCTggACTgTTCTTCACTCTTg
TBP-Z2-Cy5	(Cy5)gAgAgCCACgAACCACggCACTCATTTTC(BHQ2)
